# Dynamics of Viral Evolution and CTL Responses in HIV-1 Infection

**DOI:** 10.1371/journal.pone.0015639

**Published:** 2011-01-20

**Authors:** Yi Liu, John P. McNevin, Sarah Holte, M. Juliana McElrath, James I. Mullins

**Affiliations:** 1 Department of Microbiology, University of Washington School of Medicine, Seattle, Washington, United States of America; 2 Department of Medicine, University of Washington School of Medicine, Seattle, Washington, United States of America; 3 Department of Laboratory Medicine, University of Washington School of Medicine, Seattle, Washington, United States of America; 4 Vaccine and Infectious Disease Institute, Fred Hutchinson Cancer Research Center, Seattle, Washington, United States of America; 5 Program in Biostatistics and Biomathematics, Fred Hutchinson Cancer Research Center, Seattle, Washington, United States of America; Tsinghua University, China

## Abstract

Improved understanding of the dynamics of host immune responses and viral evolution is critical for effective HIV-1 vaccine design. We comprehensively analyzed Cytotoxic T-lymphocyte (CTL)-viral epitope dynamics in an antiretroviral therapy-naïve subject over the first four years of HIV-1 infection. We found that CTL responses developed sequentially and required constant antigenic stimulation for maintenance. CTL responses exerting strong selective pressure emerged early and led to rapid escape, proliferated rapidly and were predominant during acute/early infection. Although CTL responses to a few persistent epitopes developed over the first two months of infection, they proliferated slowly. As CTL epitopes were replaced by mutational variants, the corresponding responses immediately declined, most rapidly in the cases of strongly selected epitopes. CTL recognition of epitope variants, via cross-reactivity and *de novo* responses, was common throughout the period of study. Our data demonstrate that HIV-specific CTL responses, especially in the critical acute/early stage, were focused on regions that are prone to escape. Failure of CTL responses to strongly target functional or structurally critical regions of the virus, as well as the sequential cascade of CTL responses, followed closely by viral escape and decline of the corresponding responses, likely contribute to a lack of sustainable viral suppression. Focusing early and rapidly proliferating CTL on persistent epitopes may be essential for durable viral control in HIV-1 infection.

## Introduction

Cytotoxic T-lymphocyte (CTL) responses are associated with variable levels of HIV-1 infection control [1]–[3], yet these responses are unable to clear the virus. Viruses are able to evade CTL responses via mutations both within and flanking the epitopes [4]–[7], and selection for escape mutants is a major driving force of HIV-1 evolution [6], [8]–[10]. Once viral escape occurs, decay of corresponding CTL responses [11], [12] and generation of responses directed towards mutants and epitopes at new locations have been observed [8], [13]–[17]. Most previous studies of CTL-viral epitope dynamics examined only a few epitopes at a few time points [8], [11], [12]. Goonetilleke et al. [11] comprehensively examined the dynamics of primary HIV-1-specific CTL responses in three acutely infected subjects and found that these responses rapidly selected escape mutations, rapidly waned afterwards, and were followed by responses to epitopes that escaped more slowly or were invariant. However, the dynamics of later CTL responses and CTL responses to epitope variants were not clear.

We previously reported a comprehensive study of the first four years of HIV-1 infection in an antiretroviral therapy-naïve subject (here referred to as PIC87014) from the Seattle Primary Infection Cohort [9], [18]. We observed CTL recognition of 25 epitopes, 18 of which underwent mutational changes that, in most cases, conferred escape. Here, we studied the dynamics between CTL responses and viral evolution of these 25 epitopes and their major variants throughout the first four years of infection.

## Materials and Methods

### Clinical, virologic, immunologic, and genetic characterization of the study subject

The Institutional Review Boards for Human Subjects of the University of Washington and the Fred Hutchinson Cancer Research Center reviewed and approved this study. Written informed consent was obtained from the subject for participation in this study.

Clinical, virologic, immunologic and genetic characterization of HIV-1 infection in PIC87014 has been reported previously [9], [18], [19]. No antiretroviral therapy was administered throughout the course of study. The subject's Class I HLA alleles are A*0201, A*2501, B*1801, B*5101, C*0102, and C*1203.

Previously, viral sequences were derived from 5′ and 3′ half genomes from day 8 and from near full-length viral genomes from days 826 and 1245 after the onset of symptoms of primary infection. Targeted gene fragments were also sequenced and included *gag*-p17, *gag*-p24, *pol*-RT, *vpr*-*tat*, *vpu*, *env*-V1–V2, *env*-C2–V5, *env*-gp41, and *nef* from an additional 11 time points [22, 50, 76, 113, 155, 190, 344, 581, 769, 1035, and 1501 days post onset of symptoms of primary infection (DPS)], as well as a *gag*-p24 fragment from 414 and 491 DPS and a *gag*-p17 fragment from 491 and 667 DPS [9], [18]. The consensus viral sequence at 8 DPS was defined as the founder sequence. No new viral gene sequences were determined for this study, the previously determined sequences correspond to accession numbers DQ853426 to DQ854622 in GenBank.

CTL recognition was characterized previously by IFN-γ ELISpot assays, using cryopreserved peripheral blood mononuclear cells (PBMC) [9], [18] and 769 peptides spanning the entire HIV-1 proteome derived from the 2001 HIV-1 subtype B consensus sequence [20], as well as 169 15-mer peptides derived from this subject's founder viral proteome sequence. An assay was considered positive when the mean number of spot forming cells (SFC) for the tested peptide was at least two-fold greater than that measured for the negative control and greater than 50 SFC/10^6^ PBMC after subtraction of the mean value for the negative control. For ELISpot assay-positive 15-mers, shorter peptides were synthesized to define optimal CTL epitopes, and a total of 25 optimal epitopes were defined in this subject. CTL responses to the 25 epitopes were examined at 19 time points (8, 22, 29, 34, 50, 155, 190, 259, 304, 344, 491, 496, 680, 769, 826, 829, 1035, 1245, and 1501 DPS), with individual epitopes examined at 5 to 15 different time points [9], [18]. In addition, CTL functional avidity to an epitope was determined by measuring the half maximal effective concentration (EC_50_), the effective peptide concentration that elicited 50% of the peak IFN-γ response in serial peptide dilutions in the ELISpot assays.

**Figure 1:**
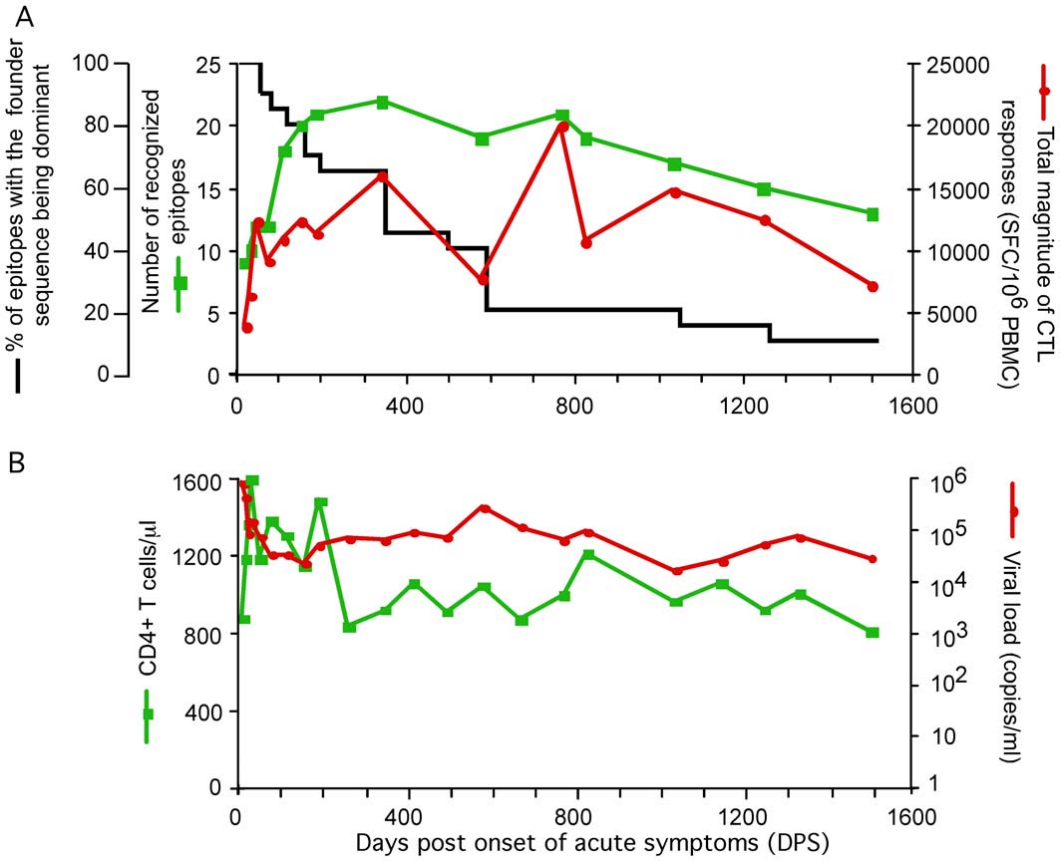
Clinical measures and summaries of CTL recognition and emergence of mutational variants in recognized epitopes. A) Twenty-five epitopes were recognized in this subject during the four-years of evaluation. CTL responses to all 25 founder epitopes were examined at 14 time points, with a subset of epitopes also examined at up to another seven time points. The number of epitopes recognized and the total magnitudes of CTL responses of the 14 time points are shown. Viral sequences encompassing 24 epitopes (not including Pol NL8, for which less data was available) were obtained from the 14 time points, including 13 at which CTL responses were measured. The percentage of epitopes of which the founder sequence was dominant (over 50%) in the sampled viral population is also shown, thus reflecting the cumulative escape of epitopes through time. B) Viral load and CD4^+^ T cell counts over time.

**Figure 2:**
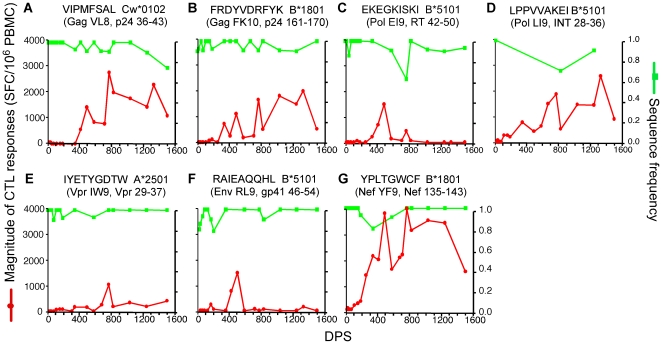
Dynamics of CTL responses to persistent viral epitopes. Longitudinal changes in the magnitudes of CTL responses and sequence frequencies of the founder epitopes. Green lines represent the sequence frequencies of the founder epitopes and red lines represent the magnitude of the corresponding CTL responses.

**Figure 3:**
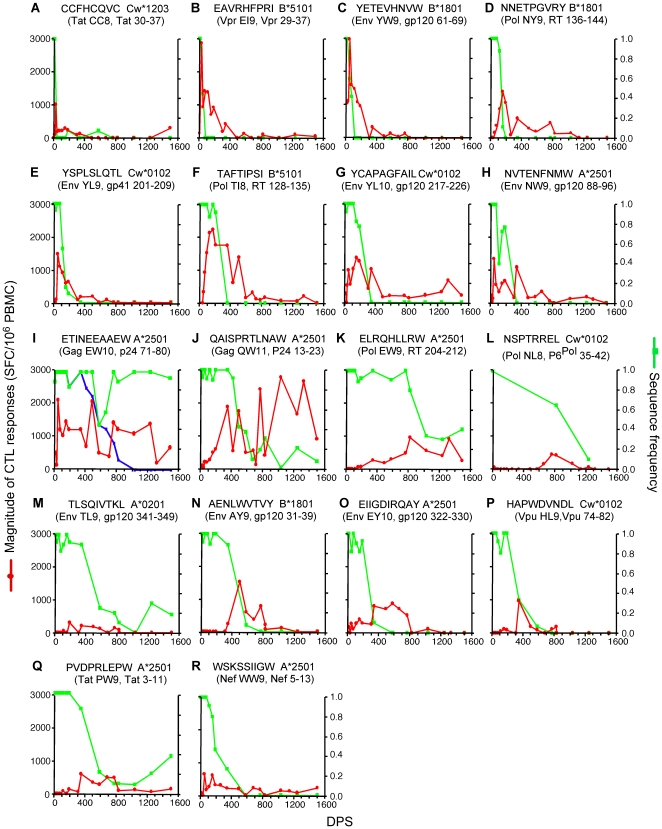
Dynamics of CTL responses to evolving viral epitopes. See legend to Figure 2 for conventions. Panels A to H, evolving epitopes experiencing strong selection; panels I to R, evolving epitopes experiencing weak selection. In panel I, the blue line represents the sequence frequency of the founder epitope with its C-terminal flanking sequence intact (ETINEEAAEWdrv, the lower case letters indicate the flanking sequence, and the underlined letter is the location of the escape mutation that impaired epitope processing).

In the current study, 568 additional IFN-γ ELISpot assays were performed so that 24 epitopes were examined at at least 13 time points from which viral sequences were previously available, and at a new time point, 34 DPS (Figures 1, 2 and 3). Viral sequences encompassing the 25^th^ epitope, Pol NL8, were only available at the three time points from which half or near full-length genome sequences were obtained (Figure 3L). CTL recognition at 8 DPS was examined for only a subset of epitopes because PBMC samples were limited. Some epitopes were also tested at one or more of the following additional time points: 16, 307, 414, 667, 713, 1144 and 1329 DPS (Figures 2 and 3). For those epitopes that were largely replaced by mutants, recognition of 21 of their major variants was also examined longitudinally. In total, 46 peptides were tested for recognition at 6 to 20 time points in the current study.

### Analysis of the dynamics of CTL responses and viral evolution

Based on sequence evolution observed within each epitope, the 25 epitopes were divided into three groups: persistent - epitopes that remained largely unchanged during the course of study (Figure 2), and strongly or weakly selected - epitopes that were largely replaced by mutants (Figure 3 and Table S1). The latter two groups were defined based on selection coefficient (*s*) values. For each evolving epitope, the effect of selection was described by a selection coefficient (*s*): log[(1−*p_t_*)/*p_t_*] = log[(1−*p*_0_)/*p*_0_]+*t*log(1+*s*) [18], [21], where *p*_0_ and *p_t_* were the frequencies of the founder epitope sequence at generation 0 and *t*, respectively. *s* was determined based on logistic regression fit of the longitudinal sequence frequency change of the founder epitope. For two epitopes, Tat CC8 and Vpr EI9, mutational variants were first detected and rose to represent 100% of the sequences over the course of only one and two time points, respectively (Figures 3A and B). Thus, *s* values for these epitopes were determined conservatively using *n*/(*n*+1) as the frequency of the founder sequence at the last time point prior to detection of the mutant(s), and 1/(*n*+1) as the frequency of the founder sequence at the first time point where the mutant(s) reached 100%, with *n* being the total number of sequences obtained at the corresponding time points. Because constant selection by CTL responses was assumed, *s* values represent the average selection that an epitope experienced over time. Epitopes with *s* values above the median for all evolving epitopes were defined as strongly selected, and epitopes with *s* values equivalent to or below the median were defined as weakly selected.

The dynamics of CTL responses and viral evolution were examined by comparing the change in the magnitudes of IFN-γ-secreting CTL responses (the mean number of spot forming cells for the tested peptide after subtraction of the mean value for the negative control) to the change in sequence frequencies of founder epitopes over time. Statistical analyses were conducted using GraphPad Prism software (version 4). Correlations were tested using the Spearman rank coefficient and categorical data were compared using the Fisher exact test.

## Results

### Overall CTL responses and escape

CTL responses to all 25 epitopes recognized in this subject were examined at 14 time points over the first four years of infection, with some epitopes examined at up to 21 time points (Figures 1A, 2 and 3). Eighteen were largely replaced by mutational variants over the first 3.4 years of infection (Figure 1A), including one epitope with an escape mutation in the flanking region (see below for details). Sixty-one percent (11/18) of these epitopes underwent major replacement within the first year of infection. The total magnitude of CTL responses against the 25 epitopes increased rapidly to over 12,000 SFC/10^6^ PBMC during the first 50 days post onset of symptoms of primary HIV-1 infection (DPS), and then fluctuated around this level.

This subject was enrolled for follow-up while in Feibig stage I of infection [22], i.e., while he was HIV-specific antibody seronegative, viral RNA positive and p24 antigen negative. His viral load dropped ∼11 fold from the peak detected at 8 DPS during the first 50 DPS (Figure 1B). Viral load stabilized at around 155 DPS with another 3.4-fold drop, and then fluctuated around 10^4^ to 10^5^ copies/ml over the next 3.7 years of follow-up, all of which was without antiretroviral therapy (Figure 1B). As expected [1], [2], [11], the rapid decrease of viral load early in infection correlated temporally with the rapid increase of the number of epitopes recognized; the number of epitopes recognized was 9 at 22 DPS, increased to 18 over the first 113 DPS, then increased more slowly to 20 at 155 DPS and to 22 around one year of infection. A decrease to 13 of the sampled peptides after four years of infection was noted at the end of the study. It should be noted that the total number of epitopes recognized and total magnitude of response in this subject was likely to be increasingly underestimated with time, with our decreasing ability to explore the reactivity of peptides from the diversifying viral population.

### CTL responses to persistent epitopes

**Figure 4:**
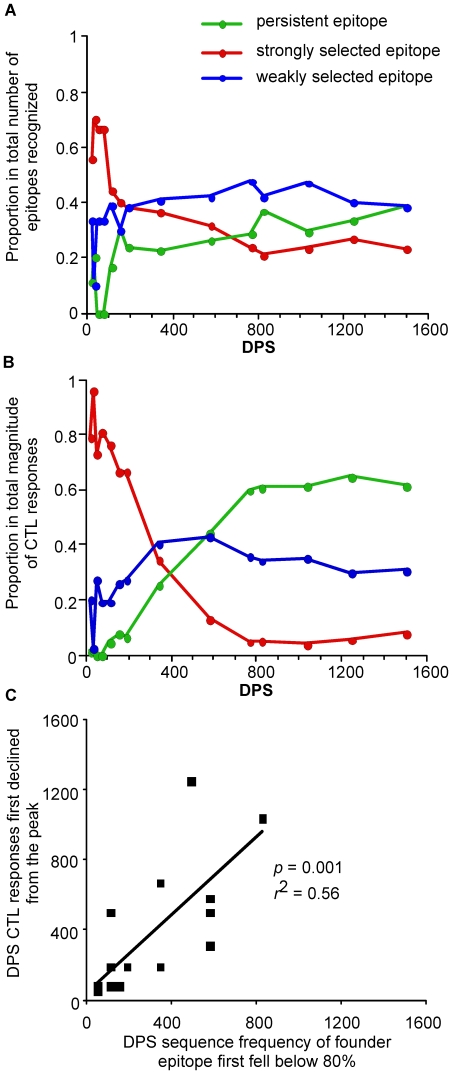
CTL recognition of persistent and evolving epitopes through time. Proportion in A) total number and B) total magnitude of CTL responses against persistent, and strongly and weakly selected evolving epitopes. Data included were from the 14 time points where CTL responses to all 25 founder epitopes were examined. C) Relationship between the emergence of epitope variants and decline of CTL responses. All 18 evolving epitopes were examined.

Seven (28%) epitopes persisted in the viral population throughout the study (Figure 2). CTL responses were detected at low levels within 50 DPS against three of these (Gag VL8, Pol LI9 and Nef YF9; Figure 2A, D and G), and within 155 DPS against the other four. Responses to all seven epitopes grew to over 1,000 SFC/10^6^ PBMC after 0.7–2 years of infection. From about six months of infection onward, 25% growing to ∼40% of the 25 epitopes recognized were persistent (Figure 4A), and by about two years of infection, CTL responses to these epitopes contributed to about 60% of the total magnitude of overall responses (Figure 4B). Responses to two persistent epitopes, Pol EI9 and Env RL9, declined to marginal or undetectable levels after two years of infection (Figure 2C and F), whereas responses to the other five persisted, and usually grew over time. Despite the decay of responses in some cases, no mutations reaching 25% in the sampled viral population were persistent within or within 10 amino acids of these epitopes.

### CTL responses to evolving epitopes

Eighteen (72%) of the epitopes detected evolved over the course of study (Figure 3). Although the founder sequence of epitope Gag EW10 (Gag p24 71–80) was mostly maintained (Figure 3I), a transient escape mutation was observed around 600 DPS that caused a significant loss in replication fitness of the virus [18], [23]. However, the escape mutant reverted back to the susceptible founder state, as the epitope acquired an amino acid substitution three amino acids C-terminal to the epitope. The latter mutation enhanced proteolytic cleavage within the epitope and thus impaired appropriate epitope processing [23]. The founder sequence encompassing EW10 and its C-terminal flanking region was eventually lost in the sampled viral population (Figure 3I, blue line).

Eight (32%) epitopes had selection coefficients above the median value of 0.035 for all evolving epitopes (Table S1, Figure 3A to H). The founder sequence of Tat CC8 declined below the detection level (i.e., were not detected in 10 to 15 clonal sequences) within 50 DPS and the founder sequences of the other seven epitopes became undetectable within about one year of infection. CTL responses were detected as early as 8 DPS against epitope Vpr EI9, and within 50 DPS against the other seven strongly selected epitopes. Peak responses to all eight epitopes exceeded 1,000 SFC/10^6^ PBMC in magnitude within 155 DPS, but the proportions in total number and magnitude of the CTL response to these epitopes quickly waned. During the first 50 DPS, when the viral load decreased most rapidly, over 50% of the epitopes recognized were strongly selected (Figure 4A), and CTL responses to these epitopes contributed 73% to 96% of the total magnitude (Figure 4B). After two years of infection, less than 30% of the epitopes recognized were strongly selected, and CTL responses to these epitopes contributed to only about 5% of the total magnitude.

For the ten (40%) epitopes experiencing weaker selection (Table S1, Figure 3I to R), founder sequences were still detectable after 1.5 years of infection. CTL responses were detected within 50 DPS against five of these epitopes (Gag EW10, Gag QW11, Pol NL8, Env EY10 and Nef WW9; Figure 3I, J, L, O and R), and within a year against the other five. In general, however, CTL responses to these epitopes were weaker, peaking at over 1,000 SFC/10^6^ PBMC for only four epitopes (Figure 2I, J, K and N). As expected, while weakly selected epitopes constituted the largest group (∼40%) of epitopes recognized by one year of infection (Figure 4A), the total magnitude of the responses directed at these epitopes were secondary to the persistent epitopes after about two years of infection (Figure 4B).

Strikingly, the emergence of epitope escape mutant(s) was immediately followed by a decline in CTL targeting that epitope. The time at which the frequency of a founder epitope first fell below 80% correlated significantly with the decline of the corresponding CTL responses from their peak (*p* = 0.001, linear regression; Figure 4C).

### The relationship between the dynamics of CTL responses and selection strength

**Figure 5:**
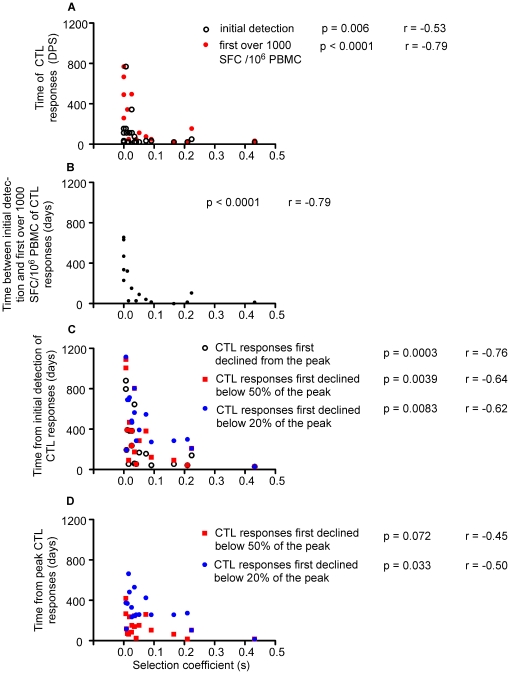
Relationship between the dynamics of CTL responses and selection strength. A) The time of the initial detection of CTL responses to an epitope, and the time of the first observation of a response over 1000 SFC/10^6^ PBMC, each as a function of *s* (selection coefficient). B) The time interval between the initial detection of CTL responses to an epitope and the first observation of magnitude over 1000 SFC/10^6^ PBMC as a function of *s* value. C) The time interval between the initial detection of CTL responses to an evolving epitope and the first observation of responses declined from the peak, below 50% and below 20% of the peak magnitude as a function of *s*. D) The time interval between the peak CTL responses to an evolving epitope and the first observation of responses declining below 50% and 20% of the peak magnitude as a function of *s*. For Gag QW11, cross-reactivity to the founder epitope from the de novo CTL responses to its mutant was observed from 769 DPS onward. Therefore, only data before 769 DPS were used for this epitope.

Earlier detection of CTL responses correlated with stronger selection, or greater *s* values (*p* = 0.006, *s* values for persistent epitopes were set to 0, Spearman rank coefficient; Figure 5A). CTL responses to all seven persistent epitopes, all eight evolving epitopes experiencing strong selection, and only three of ten evolving epitopes experiencing weak selection peaked over 1000 SFC/10^6^ PBMC. Persistent and strongly selected epitopes were more likely to peak over 1000 SFC/10^6^ PBMC than those experiencing weak selection (*p* = 0.004 for both comparisons, Fisher's exact test). For epitopes against which CTL responses peaked at over 1000 SFC/10^6^ PBMC, we found that stronger selection correlated with responses reaching this level earlier (*p*<0.0001, Spearman rank coefficient; Figure 5A) and faster (*p*<0.0001, Figure 5B).

CTL responses exerting stronger selection pressure also tended to decline earlier and faster. After initial detection of CTL responses to an evolving epitope, greater *s* values correlated with earlier observation of the first decline of CTL responses from the peak (*p* = 0.0003, Spearman rank coefficient, Figure 5C), the first decline of responses below 50% (*p* = 0.0039), and below 20% (*p* = 0.0083) of the peak magnitude. Greater *s* values also tended to correlate with shorter time intervals between the peak response to an evolving epitope and the observation of the first decline of the responses below 50% (*p* = 0.033, Figure 5D), and 20% (*p* = 0.072) of the peak magnitude.

When we examined the relationship between the dynamics of CTL responses and selection strength using data from only the 14 time points at which we evaluated all 25 epitopes, we obtained similar results (data not shown). In summary, CTL responses that exerted stronger selective pressure occurred earlier, proliferated faster, and decayed faster.

### CTL responses to epitope variants

**Figure 6:**
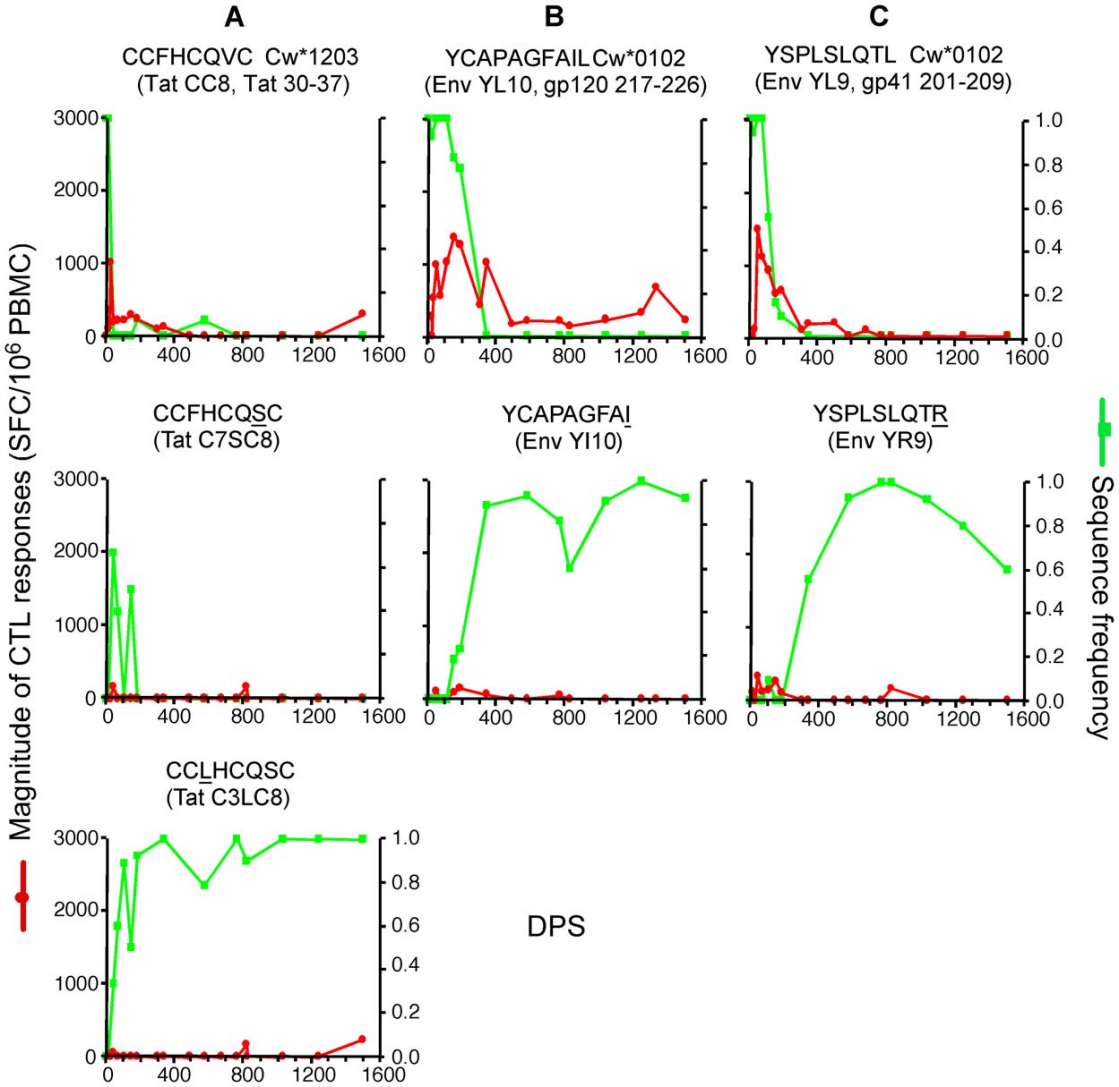
Dynamics of CTL responses to major variants of evolving epitopes. CTL responses to all major variants of these epitopes were marginal or undetectable throughout the course of study. See legend to Figure 2 for conventions. Each column of panels represent analysis of a single epitope and its major variants. The underlined amino acid corresponds to the position responsible for mutation from the founder sequence shown in the top panels.

**Figure 7:**
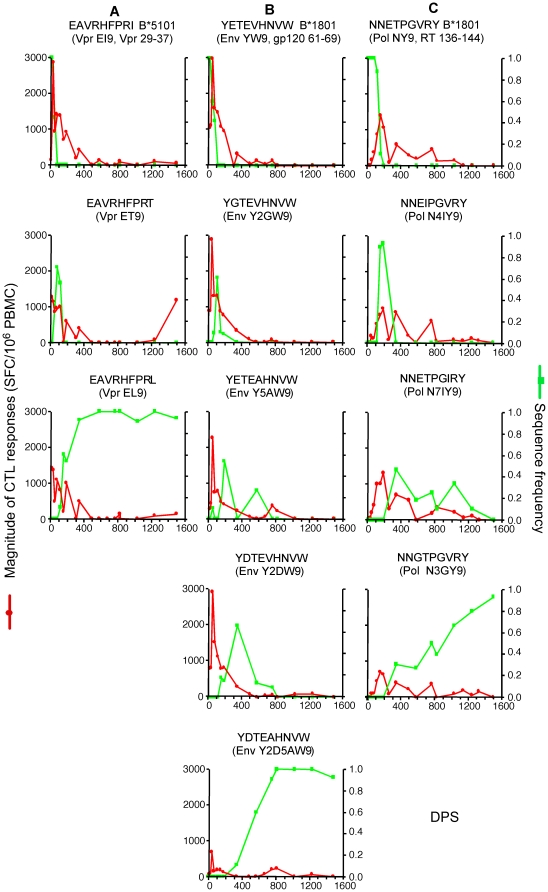
Dynamics of CTL responses to major variants of evolving epitopes. See legend to Figure 6 for conventions. CTL responses to the major variants of these epitopes were similar in magnitude over time to those observed for the founder epitopes and tended to eventually become marginal or undetectable.

**Figure 8:**
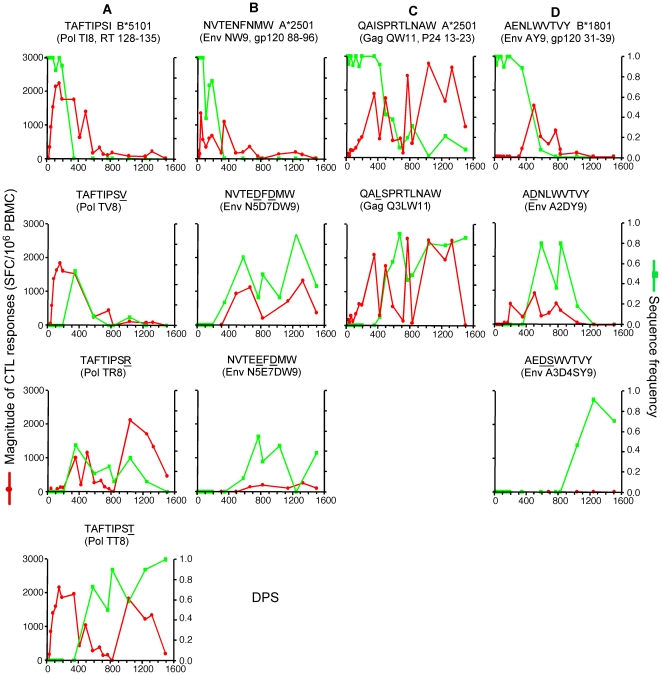
Dynamics of CTL responses to major variants of evolving epitopes. See legend to Figure 6 for conventions. Strong and specific CTL responses to some major variants of these epitopes were observed after the sequences of the founder epitopes became undetectable or minor in the sampled viral population.

We next analyzed the CTL-viral dynamics in a total of 21 major variants of 10 evolving epitopes (Figure 6, 7 and 8). We found that CTL responses to all major variants of Tat CC8, Env YL10 and Env YL9 were marginal or undetectable throughout the course of study (Figure 6). However, responses recognizing at least one major variant of the other seven epitopes were evident, peaking at levels close to or exceeding 1,000 SFC/10^6^ PBMC (Figures 7 and 8).

We observed CTL responses with high magnitudes that reacted with epitope variants even before the variant sequences were detected (or became abundant) in the sampled viral population (e.g., variants of Vpr EI9, Env YW9, Pol NY9, Pol TI8 and Gag QW11; Figures 7, 8A and 8C). These responses were similar in magnitude over time to those observed for the founder epitopes, indicating that they corresponded to cross-recognition by the responses elicited by the founder epitopes. Compared to their founder epitopes, some cross-recognized variants that were transiently dominant in the viral population (Y5AW9 of Env YW9, and N7IY9 and N4IY9 of Pol NY9, Figure 7B and C) had similar or even greater functional avidities (Table S1). However, except Q3LW11 of Gag QW11 (Figure 8C), the cross-recognized variants that were eventually nearly fixed (EL9 of Vpr EI9, Y2D5AW9 of Env YW9, N3GY9 of PolNY9, and TT8 of Pol TI8; Figures 7 and 8A) had avidities more than 10 fold lower than their founder epitopes (Table S1).

We also observed strong and sometime specific responses to epitope variants after the sequences of the founder epitopes became undetectable (variants TR8 and TT8 of Pol TI8, and N5D7DW9 of Env NW9; Figure 8A and B) or minor in the sampled viral population (variant Q3LW11 of Gag QW11; Figure 8C), indicating development of de novo responses against the variants. TR8 and N5D7DW9 were largely replaced by other variants, indicating escape from these de novo responses. The de novo responses to TR8, TT8 and N5D7DW9 recognized the founder epitopes poorly (Figure 8A and B), whereas de novo responses to Q3LW11 appeared to cross-recognize the founder QW11 (Figure 8C) with similar avidity (Table S1).

Finally, we observed recognition of some variants despite the fact that at that time, the variant sequences were not yet observed and responses to the founder epitopes and other major variants were not detected (variant ET9 of Vpr EI9 at 1501 DPS and A2DY9 of Env AY9 at 190 DPS; Figures 7A and 8D).

### An example of CTL responses with broad cross-reactivities

**Figure 9:**
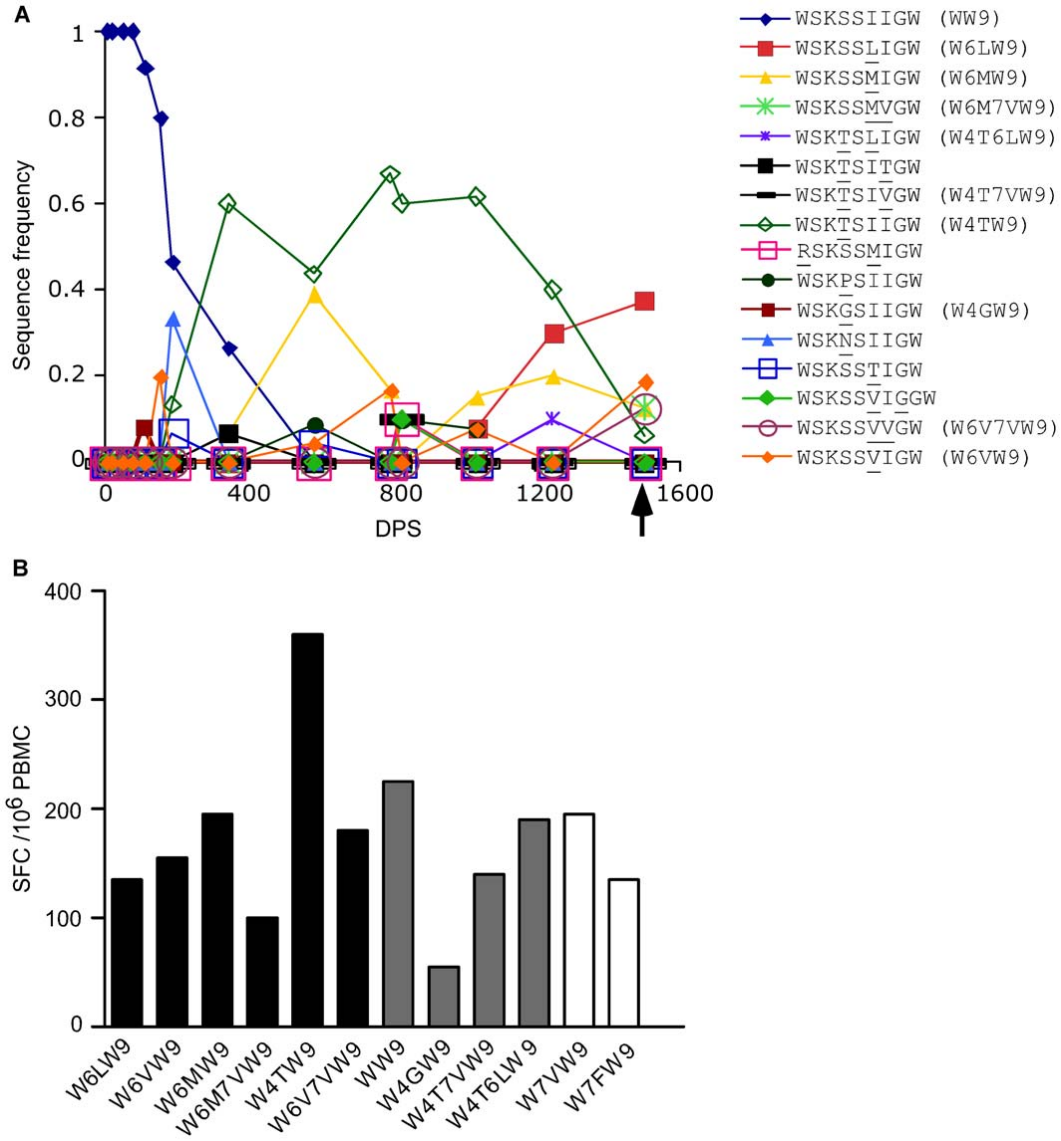
Variants of Nef WW9 recognized by CTL at 1501 DPS. A) Sequence evolution of variants of Nef WW9 observed through the course of study. The arrow indicates the time point 1501 DPS, when the CTL responses against Nef WW9 variants were examined. B) CTL responses against Nef WW9 variants at 1501 DPS. Black bars represent variants whose sequences were observed at 1501 DPS. Grey bars represent variants whose sequences were observed only before 1501 DPS. White bars represent variants whose sequences were not observed in this subject.

To further study the breath of CTL cross-reactivity, we examined CTL responses at 1501 DPS to epitope Nef WW9 and its 15 variants observed over the course of study (Figure 9A), as well as an additional four variants not detected in the subject, including the consensus subtype B sequence from the HIV Database. A total of 12 epitope variants were recognized (Figure 9B), including: All six variants whose sequences were observed at 1501 DPS (with magnitudes of responses ranging from 100 to 360 SFC/10^6^ PBMC); four variants whose sequences were observed only before 1501 DPS (with magnitudes ranging from 55 to 225 SFC/10^6^ PBMC); and two variants whose sequences were not observed in this subject (195 and 135 SFC/10^6^ PBMC). CTL responses to Nef W4TW9 (WSKTSIIGW) showed the highest magnitude, although this variant was detected in only 7.5% of the sampled viral population at 1501 DPS.

## Discussion

We comprehensively analyzed the dynamics of CTL responses and viral evolution over the first four years of HIV-1 infection of an antiretroviral therapy-naïve subject. We found that CTL responses developed sequentially and required constant antigenic stimulation for persistence. CTL responses that exerted strong selection pressure emerged and predominated early and proliferated rapidly. However, as the founder epitopes were replaced by mutational variants, the initial CTL responses immediately declined, most early and rapidly in strongly selected epitopes. Cross-recognition of epitope variants was common throughout the period of study, and de novo development of responses to epitope variants was also observed. Despite broad and strong overall CTL responses, viral load stabilized at a high level (10^4^ to 10^5^ copies viral RNA/ml plasma) in chronic infection.

The outgrowth of CTL epitope mutants is a balance between the antiviral effect of CTL responses and cost of escape mutations on viral replication fitness [18]. CTL responses to Gag EW10 developed early and proliferated rapidly, however, the epitope evolved slowly. The escape mutation in the epitope had a replication fitness cost, and another escape mutation outside the epitope was later acquired, allowing reversion of the first escape mutation and restoration of viral replication fitness [18], [23]. In contrast, most escape mutations in Env epitopes of this subject had no replication fitness cost or even increased viral fitness [23].

A beneficial effect would be expected if the early and fast proliferating CTL targeted persistent epitopes, which have been associated with more functional or structural constraints [18]. However, although CTL responses to a few persistent epitopes developed early, they proliferated slowly. We suggest that due to the immunodominant decoy effect of epitopes prone to escape, the early and fast proliferating CTL in this subject were unable to focus on persistent epitopes. This is consistent with previous findings showing that controlling CTL responses [24] and responses to conserved epitopes tend to be subdominant throughout HIV-1 infection [25].

It has been suggested that the maintenance of CTL responses during chronic infection might be dependent on the persistence of the antigen [26], [27]. Confirming this suggestion, we found that decreases in sequence frequency of the founder epitopes were associated with decreases in the magnitude of corresponding CTL responses. In contrast, CTL responses to five of seven persistent epitopes fluctuated but persisted. Although mutations did not accumulate within the other two epitopes or in their flanking regions, CTL responses to these two epitopes were lost after two years of infection. This might represent functional exhaustion (impaired ability to produce antiviral cytokines, to kill infected target cells, and to proliferate in response to antigen) or depletion of virus-specific CD8+ T cells after persistent antigen stimulation [28]–[31]. Therefore, maintenance of detectable levels of CTL responses is likely to require a delicate balance between antigen presence and prolonged antigen stimulation.

Due to the flexibility of T cell receptors (TCR), CTL can recognize slightly different but related HIV-1 epitope variants [32], [33]. Such cross-reactivity might suppress the outgrowth of mutational variants. We found that some early CTL responses could cross-recognize their variants with high magnitudes even before the variant sequences were abundant in the viral population. Some of these cross-recognized epitope variants had similar or even greater functional avidities than the founder epitopes and, as would be predicted, were only transiently dominant in the viral population. Nearly all cross-recognized epitope variants that were nearly fixed in later infection had avidities over 10-fold lower than the founder epitopes. Consistent with a previous report of an avidity threshold for killing by specific CTL [11], [34], our data suggest that cross-reactivity is more likely to suppress the growth of high avidity variants. In many cases, CTL responses to both the founder and the variant epitopes disappeared as the founder sequence became undetectable. Therefore, despite cross-reactivity, stimulation from the variants could not maintain the responses elicited by the founder epitopes. We also observed broad but low magnitude CTL recognition of epitope variants during later infection. Evolving epitopes in this subject travelled multiple evolutionary pathways to evade specific CTL killing, thus, although CTL cross-reactivity could effectively suppress some epitope variants, they were unable to suppress others. Cross-recognition might represent an over-activated immune system unsuccessfully catching up with the evolving viruses and a profound distraction of the immune system from targeting persistent epitopes. Failure of CTL responses to focus on critical regions of the virus, emergence of a cascade of viral escape mutants and decline of the corresponding responses may have made the antiviral effect of CTL responses unsustainable. Directing early and rapidly proliferating CTL to persistent epitopes is likely to be essential for durable viral control in HIV-1 infection.

## Supporting Information

Table S1 **Evolving epitopes and their evolving major variants.**
(DOC)
