# Supplementary material for: Dynamics of Viral Evolution and CTL Responses in HIV-1 Infection
Source: PLoS One. 2011 Jan 20;6(1):e15639. doi: 10.1371/journal.pone.0015639 (PMC3024315; doi:10.1371/journal.pone.0015639)
Supplement: Table S1 — Evolving epitopes and their evolving major variants. (DOC) [file pone.0015639.s001.doc]

# Table S1. Evolving epitopes and their evolving major variants

|  |  |  | EC_50_ | | |  |
| --- | --- | --- | --- | --- | --- | --- |
| Epitope sequence (name)*^a^* | HIV-1 protein (epitope position [aa])*^b^* | Mutant sequence (name)*^c^* | Epitope (nM)*^d^* | Mutant (nM)*^e^* | ratio*^f^* | Selection coefficient, *s* (95% CI)*^g^* |
| CCFHCQVC (Tat CC8) | Tat (30-37) | CCFHCQSC (C7SC8) | 212 | NR | F | 0.432*^h^* |
|  |  | CCLHCQVC (C3LC8) |  | NR | F |  |
| EAVRHFPRI (Vpr EI9) | Vpr (29-37) | EAVRHFPRT (ET9) | 30 | 1280 | 43 | 0.21*^h^* |
|  |  | EAVRHFPRL (EL9) |  | 550 | 18 |  |
| NNETPGVRY (Pol NY9) | RT (136-144) | NNEIPGVRY (N4IY9) | 20 | 32 | 1.6 | 0.224 (0.076-0.391) |
|  |  | NNETPGIRY (N7IY9) |  | 0.3 | 0.015 |  |
|  |  | NNGTPGVRY (N3GY9) |  | 440 | 22 |  |
| YETEVHNVW (Env YW9) | gp120 (61-69) | YGTEVHNVW (Y2GW9) | 12 | 267 | 22 | 0.165 (0.078-0.259) |
|  |  | YETEAHNVW (Y5AW9) |  | 0.07 | 0.006 |  |
|  |  | YDTEVHNVW (Y2DW9) |  | 154 | 13 |  |
|  |  | YDTEAHNVW (Y2D5AW9) |  | >1000 | >83 |  |
| YSPLSLQTL (Env YL9) | gp41 (201-209) | YSPLSLQTR (YR9) | ND | ND | ND | 0.091 (0.053-0.130) |
| TAFTIPSI (Pol TI8) | RT (128-135) | TAFTIPSV (TV8) | 19 | >2,000 | >100 | 0.073 (0.030-0.118) |
|  |  | TAFTIPSR (TR8) |  | >2,000 | >100 |  |
|  |  | TAFTIPST (TT8) |  | >2,000 | >100 |  |
| YCAPAGFAIL (Env YL10) | gp120 (217-226) | YCAPAGFAII (YI10) | 66 | NR | F | 0.051 (0.024-0.079) |
| NVTENFNMW (Env NW9) | gp120 (88-96) | NVTEDFDMW (N5D7DW9) | 1340 | NR | F | 0.041 (0.022-0.061) |
|  |  | NVTEEFDMW (N5E7DW9) |  | NR | F |  |
| WSKSSIIGW (Nef WW9) | Nef (5-13) |  |  |  |  | 0.035 (0.022-0.048) |
| EIIGDIRQAY (Env EY10) | gp120 (322-330) |  |  |  |  | 0.035 (0.020-0.050) |
| HAPWDVNDL (Vpu HL9) | Vpu (74-82) |  |  |  |  | 0.027 (0.017-0.037) |
| AENLWVTVY (Env AY9) | gp120 (31-39) | ADNLWVTVY (A2DY9) | 37 | 15 | 0.45 | 0.026 (0.017-0.036) |
|  |  | AEDSWVTVY (A3D4SY9) |  | NR | F |  |
| PVDPRLEPW (Tat PW9) | Tat (3-11) |  |  |  |  | 0.019 (0.013-0.026) |
| ETINEEAAEW (Gag EW10) | p24 (71-80) |  |  |  |  | 0.016 (0.011- 0.020)*^i^* |
| QAISPRTLNAW (Gag QW11) | P24 (13-23) | QALSPRTLNAW (Q3LW11) | 500 | 1200 | 2.4 | 0.012 (0.008-0.015)*^j^* |
| TLSQIVTKL (Env TL9) | gp120 (341-349) |  |  |  |  | 0.009 (0.006-0.011) |
| ELRQHLLRW (Pol EW9) | RT (204-212) |  |  |  |  | 0.007 (0.004-0.009) |
| NSPTRREL (Pol NL8) | p6^Pol^ (35-42) |  |  |  |  | 0.007 (0.002-0.012) |

*^a^* Founder amino acid sequences of recognized CTL epitopes. Epitope sequences correspond to the autologous viral sequences obtained from the first time point, 8 DPS.

*^b^* HXB2 amino acid numbering used.

*^c^* Mutated amino acids are underlined.

*^d^* The effective concentration of half-maximal binding (EC_50_) was measured at the time point at which the epitope elicited peak CTL responses. The EC_50_ was determined by peptide titration and generation of a dose-response curve.

*^e^* ND, not determined. NR, no response, i.e., CTL recognition of the mutational variant was not detected by ELISpot assays (<50 SFC/10^6^ PBMC, or no more than two-fold of that measured for the negative control) and the EC_50_ of the mutational variant was not measured.

*^f^* EC_50_ ratio = EC_50_ of mutational variant /EC_50_ of founder epitope. F, full escape, i.e., no CTL recognition of the mutational variant was detected.

*^g^* Selection coefficient, *s* values were determined using logistic regression.

*^h^* For epitopes Tat CC8 and Vpr EI9, there was a lack of data during the short interval between detection and fixation of the mutational variants. The *s* values for these epitopes were determined conservatively using *n*/(*n*+1) as the frequency of the founder epitope sequence at the last time point prior to detection of the mutational variants, and 1/(*n*+1) as the frequency of the founder epitope sequence at the first time point where only the mutational variants were observed, with *n* being the total number of sequences obtained at the corresponding time points.

*^i^*For Gag EW10, the *s* value was determined by the sequence frequency change of ETINEEAAEWdrv (the lower case letters indicating the flank sequence), because the mutation at the flanking valine (underlined) impaired appropriate epitope processing .

*^j^* For Gag QW11, cross-reactivity to the founder epitope from the de novo developed CTL responses to its mutant was observed from 769 DPS onward. To minimize the interference from the cross-reactivity, we also calculated the *s* value using only data before 769 DPS and obtained *s* = 0.020 with 95% CI being 0.013 to 0.027.
